# Lutetium-177-PSMA-617: A Vision of the Future

**DOI:** 10.1080/15384047.2022.2037985

**Published:** 2022-02-27

**Authors:** Elias Chandran, William D. Figg, Ravi Madan

**Affiliations:** Genitourinary Malignancies Branch, Center for Cancer Research, National Cancer Institute, National Institutes of Health, Bethesda, MD, USA

**Keywords:** Prostate cancer, mCRPC, lutetium, PSMA, radioligand therapy, FDG PET-CT, cabazitaxel

## Abstract

In the last decade, many life-prolonging therapeutic options have emerged for metastatic castration-resistant prostate cancer (mCRPC). The recent VISION trial is the first to demonstrate a survival benefit of Lutetium-177[^177^Lu]Lu-PSMA-617 in post-chemotherapy mCRPC. This journal club reviews the VISION trial in the context of the earlier TheraP trial of [^177^Lu]Lu-PSMA-617 in mCRPC post docetaxel and androgen pathway inhibition, to provide direction for the real-world application of [^177^Lu]Lu-PSMA-617. Treatment in the control groups differed significantly between both trials and may have influenced outcomes: TheraP mandated cabazitaxel whereas VISION’s design could not allow it. In both trials, [^177^Lu]Lu-PSMA-617 had a good safety profile, with common adverse events being fatigue, nausea, dry mouth, marrow suppression and diarrhea. Given its efficacy and favorable safety even in heavily pre-treated patients, [^177^Lu]Lu-PSMA-617 provides hope to mCRPC patients and may be applied to earlier disease stages in future investigations.

## Introduction

Metastatic castration-resistant prostate cancer (mCRPC) has a poor prognosis, with an expected median survival of only 2–3 years. In the last decade alone, many new life-prolonging therapeutic options for this disease have emerged.^1,2^ A relatively novel treatment concept for this disease is targeted radioligand therapy, whereby a radioisotope is paired with a monoclonal antibody against a cancer-specific antigen, such as prostate-specific membrane antigen (PSMA), providing an elegant method of targeted radiation to the cancer whilst minimizing effects on normal tissue. Several radioisotopes have been used in radioligand therapy for mCRPC, including alpha particle emitters such as Actinium-225 and beta emitters such as Lutetium-177; however, most of the randomized clinical evidence and experience for PSMA-based radioligand therapy exists for Lutetium-177[^177^Lu]Lu-PSMA-617.^2–6^

## Clinical Trials Examining Lutetium-177[^177^Lu]Lu-PSMA-617 in Prostate Cancer

The VISION trial, recently published in the New England Journal of Medicine, is the first phase 3 trial to prove a survival benefit of [^177^Lu]Lu-PSMA-617 in the treatment of mCRPC, leading to it being granted breakthrough therapy designation (BTD) by the US Food and Drug Administration (FDA).^7,8^ This will undoubtedly raise its profile and drive its dissemination in North America, where it is currently not widely available.

Propelled by an earlier succession of pioneering clinical trials conducted at Melbourne’s Peter MacCallum Cancer Center on patients with heavily pre-treated mCRPC, [^177^Lu]Lu-PSMA-617 has already become adopted into clinical practice in Australia. In 2018, the single-arm phase 2 LuPSMA trial impressively reported PSA declines of ≥50% (PSA50 responses) in 57% of its 30 participants.^9^ Earlier this year, the randomized phase 2 TheraP trial reported PSA50 responses in 66% of its [^177^Lu]Lu-PSMA-617 group compared to 37% of its cabazitaxel group and superior progression-free survival (PFS) in the [^177^Lu]Lu-PSMA-617 group.^10^ There is also considerable experience in the use of [^177^Lu]Lu-PSMA-617 in Germany and the rest of Europe, where regulatory approvals have been in place since 2013.^11–14^

This article aims to compare the VISION trial with TheraP to provide context for the real-world application of [^177^Lu]Lu-PSMA-617. VISION is an open-label phase 3 trial which recruited men with mCRPC who had previously received at least one androgen pathway inhibitor (API) and up to two previous taxane regimens. Participants were required to have PSMA-positive mCRPC, defined as having at least one PSMA-positive metastatic lesion and no PSMA-negative lesions on gallium-68 [^68^Ga]Ga-PSMA-11 PET-CT (PSMA PET-CT). Other key inclusion criteria included ECOG performance status 0–2 and life expectancy of at least 6 months. The protocol designated two arms: an experimental arm, to receive standard care plus [^177^Lu]Lu-PSMA-617 and a control arm, to receive standard care alone. Standard care was at the physician’s discretion, but could not include cytotoxic chemotherapy, radium-223, immunotherapy, or olaparib due to the lack of safety data of combining [^177^Lu]Lu-PSMA-617 with these agents. Eight hundred and thirty-one patients were randomized 2:1 to the experimental and control arms, respectively. Both arms were well balanced with respect to median PSA levels, sites of disease and other biochemical prognostic features such as alkaline phosphatase and lactate dehydrogenase levels. There was a slightly higher proportion of patients who received more than one prior API in the control arm (54.3% versus 45.9%). The median age was about 71 years (range 40 to 94 years) and 92.4% of the participants were ECOG 0–1. After a median follow-up of 20.9 months, the experimental arm was found to have a significantly longer median radiographic PFS (rPFS) of 8.7 versus 3.4 months among 581 evaluable patients (hazard ratio (HR) 0.4, 99.2% confidence interval (CI) 0.29–0.57, p < .001) and a median OS of 15.3 vs 11.3 months among all 831 patients (HR 0.62, 95% CI 0.52–0.74, p < .001).^7^

Both TheraP and VISION recruited men with mCRPC but with slight differences. TheraP recruited men who would have been candidates for cabazitaxel as their next line of treatment: men who had prior docetaxel for mCRPC; 91% of whom received at least one prior API. It designated its control group to receive cabazitaxel based on findings from the 2019 CARD trial, which demonstrated a significant survival benefit of cabazitaxel over the alternate API in post-docetaxel mCRPC patients who had previously received abiraterone or enzalutamide.^15^ In the VISION trial, all patients must have received an API plus at least one prior taxane regimen. Its design of combining [^177^Lu]Lu-PSMA-617 with standard care in the experimental arm meant that the choices for protocol-permitted standard care were restricted in its control arm. Notably, 61.8% of its control arm were cabazitaxel-naïve, but could not receive this while on protocol. No protocol changes were made to allow its control group to receive cabazitaxel after the CARD trial was published in 2019; however, physicians had the discretion to discontinue protocol treatment if patients were deemed appropriate for chemotherapy.^7^ The inability of this significant proportion of patients to receive cabazitaxel on protocol may have been the reason for the observed survival difference between the two arms of the VISION trial.

Another major methodological difference in the Australian trials is the utilization of 2-Flourine-18[18F]fluoro-2-deoxy-D-gluycose (FDG) PET-CT scans in screening to ensure that only patients with disease concordant across both FDG and PSMA PET-CT scans were recruited.^9,10^ In the TheraP trial, 51 out of 291 (17.5%) screened patients were excluded on this basis with the rationale that FDG-avid areas with low PSMA expression are unlikely to benefit from a therapy that is highly targeted toward PSMA.^10,16^ Patients with this imaging phenotype seem to have a poor outcome: in an observational study of sixteen excluded patients from the earlier LuPSMA study with low PSMA expression or discordant FDG-avid disease, median overall survival was 2.5 months compared to 13.5 months for the patients who were included in the trial, likely reflecting end-stage, heavily pretreated disease and advanced tumor heterogeneity.^9,17^ The VISION trial did not employ FDG PET-CT scans in the screening process; however, excluded patients with metastatic lesions identified on CT which were PSMA-negative. Out of the 1003 patients who underwent PSMA PET-CT screening, 123 (12%) were excluded for this reason. It is possible that there were patients included in VISION who would have had FDG-avid disease that was occult on CT as found in the Australian trials.^9,10,17^ There is also a difference in the definition of PSMA-positive disease employed in both trials. TheraP defined it as a maximum standardized uptake value (SUV_max_) of ^68^Ga-PSMA-11 of ≥20 at a site of disease and >10 at all other measurable sites, whereas the VISION trial defined it as uptake in one or more metastases greater than that of liver parenchyma;^7,10^ however, it is unlikely that this slight difference will account for significant disparities in the results.

The clinical endpoints in both trials were slightly different. VISION was initially designed with a single primary endpoint of OS; however, the protocol was amended after about a year to include rPFS. Secondary endpoints include ORR, DOR, PFS and PSA50 response.^7^ TheraP, being a phase 2 trial, designated PSA50 response as its primary endpoint, with secondary endpoints including PFS by PCWG3 and rPFS.^10^ The efficacy outcomes of TheraP and VISION are presented in Table 1; however, direct comparison between these trials should not be made due to differences in population, sample size and methodology.

**Table 1.** Efficacy outcomes of TheraP and VISION

Endpoint	TheraP		VISION	
Arm	Lu-PSMA (n = 99)	Cabazitaxel (n = 101)	Lu-PSMA/SC (n = 551)	SC (n = 280)
mOS (m)	NA	NA	15.3	11.3
			*HR 0.62 (95% CI 0.52–0.74), p < .001*	
Median rPFS (m)	5.1 (n = 99)	5.1 (n = 101)	8.7 (n = 385)	3.4 (n = 196)
	*HR 0.63 (95% CI 0.46–0.86), p = .0028*		*HR 0.4 (99.2% CI 0.29–0.57), p < .001*	
PSA50 response (%)	66 (65/99) *(95% CI 56–75)*	37 (37/101) *(95% CI 27–46)*	46 (177/385)	7.1 (14/196)
	*p < .0001*		*OR 11.19 (95% CI 6.25–20.04)*	
ORR (%)	49 (18/37) *(95% CI 33–65)*	24 (10/41) *(95% CI 11–38)*	29.8 (95/319)	1.7 (2/120)
	*RR 2.12 (95% CI 1.10–4.08), p = .019*		*OR 24.99 (95% CI 6.05–103.24), p < .001*	
CR (%)	0 (0/37)	0 (0/41)	5.6 (18/319)	0 (0/120)

Lu-PSMA = [^177^Lu]Lu-PSMA-617, mOS = median overall survival, m = months, NA = not available, HR = hazard ratio, CI = confidence interval, rPFS = radiographic progression-free survival, PSA50 response = proportion of patients with PSA reduction ≥50%, OR = odds ratio, ORR = overall response rate, RR = relative risk, CR = complete response

In both TheraP and VISION, [^177^Lu]Lu-PSMA-617 was well-tolerated, with common treatment-related adverse events (AEs) being fatigue, nausea, dry mouth, dry eyes, anemia, thrombocytopenia, leukopenia and diarrhea, as shown in Table 2.^7,10^ [^177^Lu]Lu-PSMA-617 commonly causes dry mouth as it is taken up by the salivary gland. This occurred only at grade 1 and 2 in both trials. The experience with [^177^Lu]Lu-PSMA-617 so far has shown that the associated dry mouth is mild and likely transient in contrast to that experienced by patients who receive high doses of external beam radiation for head can neck cancers.^16,18–20^ Efforts to mitigate this AE with various strategies such as cooling have not shown success.^19^ [^177^Lu]Lu-PSMA-617 is also taken up by the lacrimal glands and there was, interestingly, a much higher rate of dry eyes reported in TheraP (29.6%) than VISION (3%). The kidneys also have high PSMA expression; however, both TheraP and VISION, and other studies of [^177^Lu]Lu-PSMA-617 have mostly not demonstrated significant treatment-related nephrotoxicity,^7,10,12,14,20^ which is a very rare occurrence.^18^ Health-related quality of life (HRQoL) data from VISION has not been reported in detail, but preliminary data shows more favorable patient reported outcomes (PROs) in the [^177^Lu]Lu-PSMA-617 arm. Similarly, the TheraP trial reported similar or better PROs in the [^177^Lu]Lu-PSMA-617 arm compared to cabazitaxel.

**Table 2.** Selected Adverse Events from TheraP and VISION

Adverse Event	TheraP		VISION	
Arm	Lu-PSMA (n = 98)	Cabazitaxel (n = 85)	Lu-PSMA/SC (n = 529)	SC (n = 205)
Fatigue (%) Grade 1–2 (Grade ≥3)	70.4 (5.1)	71.8 (3.5)	37.2 (5.9)	21.5 (1.5)
Nausea (%) Grade 1–2 (Grade ≥3)	39.8 (1.0)	34.1 (0)	34.0 (1.3)	16.1 (0.5)
Dry mouth (%) Grade 1–2 (Grade ≥3)	60.2 (0)	21.2 (0)	38.8 (0)	0.5 (0)
Dry eyes (%) Grade 1–2 (Grade ≥3)	29.6 (0)	3.5 (0)	3.0 (0)	1.0 (0)
Anemia (%) Grade 1–2 (Grade ≥3)	19.4 (8.2)	12.9 (8.2)	18.9 (12.9)	8.3 (4.9)
Thrombocytopenia (%) Grade 1–2 (Grade ≥3)	18.4 (11.2)	4.7 (0)	9.3 (7.9)	3.4 (1)
Leukopenia (%) Grade 1–2 (Grade ≥3)	10.2 (1.0)	5.9 (1.2)	10.0 (2.5)	1.5 (0.5)
Neutropenia (%) Grade 1–2 (Grade ≥3)	7.1 (4.1)	4.7 (12.9)	5.1 (3.4)	1.0 (0.5)
Diarrhea (%) Grade 1–2 (Grade ≥3)	18.4 (1.0)	51.8 (4.7)	18.1 (0.8)	2.4 (0.5)
Increased serum creatinine (%) Grade 1–2 (Grade ≥3)	4.1 (0)	2.4 (0)	5.1 (0.2)	2.0 (0.5)

Lu-PSMA = [^177^Lu]Lu-PSMA-617, SC = standard care

## The Real World

Retrospective studies on unselected real-world patients with mCRPC have shown comparable efficacy and safety of [^177^Lu]Lu-PSMA-617.^12,20–22^ Notably, the rate of FDG PET-CT use was not reported in these studies.^12,20,21^ Due to the observation of rare long-term hematological AEs in patients with neuroendocrine tumors after the use of ^177^Lutetium in peptide receptor radionuclide therapy (PRRT),^23,24^ a German study focused on [^177^Lu]Lu-PSMA-617 associated myelosuppression. Their data of 140 heterogeneous patients with mCRPC showed acceptable rates of grade ≥3 anemia (7.1%), thrombocytopenia (4.3%) and leukopenia (3.6%). The myelosuppression was frequently reversible and risk factors identified for significant myelosuppression were high bone tumor burden, previous taxane chemotherapy and baseline cytopenia. At median follow-up of 8 months, no cases of late-onset severe myelosuppression or myelodysplastic syndrome were reported.^25^ Another study showed hematological safety of [^177^Lu]Lu-PSMA-617 in patients with high bone tumor burden.^22^

An international study, NIGHTCAP, is currently prospectively collecting data on patients who receive [^177^Lu]Lu-PSMA-617 worldwide. Aiming to include eligible patients with prostate cancer from anywhere in the world, it has a basic protocol that is adaptable to physician discretion and evolving best practice. This study will be highly valuable in reporting outcomes of [^177^Lu]Lu-PSMA-617 on a large scale in unselected real-world patients.^26^

Whether or not using only PSMA PET-CT to screen for [^177^Lu]Lu-PSMA-617 treatment reflects practice in the real world is unknown; however, this is most likely the case for one simple reason: cost. Outside of a clinical trial setting, [^177^Lu]Lu-PSMA-617 is likely reserved for the last line of treatment in heavily pre-treated patients who have the financial means to self-fund it. For a self-paying patient, a FDG PET-CT scan would be a significant addition to the already substantial cost of [^177^Lu]Lu-PSMA-617 treatment. Furthermore, these patients are likely to be highly motivated to pursue the treatment regardless of FDG PET-CT findings.

For healthcare systems, widespread adoption of [^177^Lu]Lu-PSMA-617 will pose significant challenges. Besides the high costs involved, there are important logistical considerations. Given the demonstrated efficacy and regulatory approvals, there will likely be a surge in demand for [^177^Lu]Lu-PSMA-617 treatment, which could overwhelm existing treatment facilities and specialist staffing levels, and may outstrip the limited capacity for radioligand production, as was seen with radium-223 in 2014, the year after it gained FDA approval.^27,28^ In countries with public healthcare systems, there may be longer delays due to the need for high-level funding decisions based on benefit-to-cost assessments.^27^ However, these are mostly teething issues which will be overcome with time and experience.

## Conclusion

There are refinements to be made on the best method of selecting mCRPC patients for [^177^Lu]Lu-PSMA-617; however, we have an excellent starting point: PSMA PET-CT scans. The use of FDG PET-CT scans in screening for this treatment modality does not appear to be widely adopted, and despite this, efficacy does not seem to be affected. There is also the question of determining where [^177^Lu]Lu-PSMA-617 is best placed in the increasingly complex treatment sequence; however, this will undoubtedly be clarified as more studies are completed. Despite these challenges, [^177^Lu]Lu-PSMA-617 is a beacon of hope for patients with mCRPC due to its impressive efficacy even in heavily pre-treated patients and its low toxicity. Exuberance exists for its use in earlier stages of prostate cancer such as in metastatic castration-sensitive disease and in biochemically recurrent disease; however, close monitoring of long-term AEs and HRQoL impact will be needed to justify its use in these patients due to their significantly longer survival.

## Declarations

R Madan receives research funding from Bayer AG. The authors make no other declarations.
